# A Telehealth-Delivered Pulmonary Rehabilitation Intervention in Underserved Hispanic and African American Patients With Chronic Obstructive Pulmonary Disease: A Community-Based Participatory Research Approach

**DOI:** 10.2196/13197

**Published:** 2020-01-31

**Authors:** Renee Pekmezaris, Andrzej Kozikowski, Briana Pascarelli, Gisele Wolf-Klein, Eugenia Boye-Codjoe, Sonia Jacome, Danielle Madera, Donna Tsang, Brenda Guerrero, Richard Medina, Jennifer Polo, Myia Williams, Negin Hajizadeh

**Affiliations:** 1 Northwell Health Manhasset, NY United States; 2 Department of Medicine Hofstra Northwell School of Medicine Manhasset, NY United States; 3 Department of Occupational Medicine, Epidemiology, and Prevention Hofstra Northwell School of Medicine Great Neck, NY United States

**Keywords:** COPD, pulmonary rehabilitation, telehealth, CPBR, disparities, telemonitoring

## Abstract

**Background:**

Although home telemonitoring (TM) is a promising approach for patients managing their chronic disease, rehabilitation using home TM has not been tested for use with individuals living with chronic obstructive pulmonary disease (COPD) residing in underserved communities.

**Objective:**

This study aimed to analyze qualitative data from focus groups with key stakeholders to ensure the acceptability and usability of the TM COPD intervention.

**Methods:**

We utilized a community-based participatory research (CBPR) approach to adapt a home TM COPD intervention to facilitate acceptability and feasibility in low-income African American and Hispanic patients. The study engaged community stakeholders in the process of modifying the intervention in the context of 2 community advisory board meetings. Discussions were audio recorded and professionally transcribed and lasted approximately 2 hours each. Structural coding was used to mark responses to topical questions in interview guides.

**Results:**

We describe herein the formative process of a CBPR study aimed at optimizing telehealth utilization among African American and Latino patients with COPD from underserved communities. A total of 5 major themes emerged from qualitative analyses of community discussions: equipment changes, recruitment process, study logistics, self-efficacy, and access. The identification of themes was instrumental in understanding the concerns of patients and other stakeholders in adapting the pulmonary rehabilitation (PR) home intervention for acceptability for patients with COPD from underserved communities.

**Conclusions:**

These findings identify important adaptation recommendations from the stakeholder perspective that should be considered when implementing in-home PR via TM for underserved COPD patients.

**Trial Registration:**

ClinicalTrials.gov NCT03007485; https://clinicaltrials.gov/ct2/show/NCT03007485

## Introduction

### Background

Chronic obstructive pulmonary disease (COPD) is the leading cause of hospitalization for older adults in the United States. According to the World Health Organization, COPD will become the third leading cause of death worldwide by 2020 [1,2]. Disparity populations unquestionably bear a more significant burden of suffering, with death rates rising faster than that in whites [1,2]. African Americans and Hispanics are disproportionately affected by social and economic inequalities that impact access to care, including language, acculturation, and immigration status [3]. Both groups bear a high burden of illness and death due to COPD and asthma and are twice as likely to visit the emergency room for COPD-associated conditions as compared with non-Hispanic whites [4,5]. Higher rates of smoking, reduced health access (especially to pulmonary rehabilitation [PR]), and lower socioeconomic status (SES) all contribute to this high disease burden [6,7]. Lower SES and ethnic minority COPD patients are also at increased risk for readmission. COPD patients admitted for COPD exacerbation have a 23% and 50% risk of 30-day and 12-month readmission [8], respectively, and African American and Hispanic race or ethnicity is associated with an almost twofold increase in hospitalization risk [9]. Patients and their caregivers suffer from discontinuity of care and decreased quality of life with each of these transitions into and out of the hospital. This phenomenon has been adopted as a marker of quality care and is tied to penalties and incentives imposed by large payers, including the Centers for Medicare and Medicaid described in more detail below.

Fortunately, early PR after admission has been shown to improve patient quality of life, satisfaction, and adherence; decrease hospitalization; and improve functional capacity, as measured by the COPD Assessment Test; body mass index, airflow obstruction, dyspnea, and exercise capacity index; 2-min step test; and 6-min walk test [10,11]. Unfortunately, referral and uptake rates are poor, particularly for African American and Hispanic patients, with only a small proportion of the intended target population receiving PR [10,12,13].

Previous research suggests that interventions that have been culturally tailored may result in improved patient outcomes. To improve generalizability across racial, ethnic, and income groups, COPD interventions should be tailored for acceptability and relevance for at-risk, underserved populations [14-16]. The community-based participatory research (CBPR) approach is one such approach to cultural tailoring. CBPR, a collaborative approach between researchers and stakeholders (including patients, caregivers, community-based organizations, and providers), has been applied in studies to assist in developing programs that address the needs of underserved communities in multiple areas [14]. These areas include heart failure [17], mental health [18], cancer [19], sexually transmitted infections [20-22], and smoking [23]. Similar to a science practitioner model, the purpose of the CBPR process is to address gaps between theoretical and *real-world* implementation using joint decision making. These decisions can include defining the research question, collecting and analyzing the data, interpreting the findings, and disseminating the results [14].

### Objectives

There is a dearth of literature regarding telehealth adaptation in COPD patients from underserved communities. The qualitative process reported herein describes the method used to identify factors perceived by community members to be crucial in successfully testing a home telemonitoring (TM) program in communities with poor access to care. We used a CBPR approach via a community advisory board (CAB) that included African American and Hispanic COPD patients and caregivers, community advocates, and disparity experts in the New York metropolitan area. This paper presents community and stakeholder feedback on iterative adaptations to a research study testing telehealth-delivered PR. Involving the community in outcome selection, intervention adaption, and interpretation of results is key in enabling overall intervention effectiveness, as one that is not acceptable in a particular community is not be likely to be replicated [16].

This study aimed to explore the perspectives of community stakeholders through the conduct of 2 focus groups of members of a CAB established to ensure patient centeredness at every phase of a mixed methods, comparative effectiveness research study of in-home telepulmonary rehabilitation.

## Methods

### Study Design

Data shown herein were collected during the first 2 of the 5 CAB meetings that occurred during the first 2 years of the 3-year study period. These initial 2 CAB meetings were conducted before the randomized controlled trial (RCT) to culturally tailor the intervention to the needs of the community. Different moderator guides were developed for each of the CAB meetings (see Multimedia Appendix 1) [24].

### Participants

Approximately 20 CAB members were invited to each meeting, with about one-third representing patients and caregivers, one-third representing providers (pulmonologists, researchers, and primary care physicians), and another one-third representing the other stakeholders (such as community-based organizations). Meetings were held in a private room of a public library in Queens, New York, to facilitate attendance by community members. The vast majority of CAB members attended both meetings (CAB meeting 1=10 and CAB meeting 2=18). CAB members were reimbursed (US $50) for their time and transportation for participation in each discussion.

CAB members included stakeholders, including African American and Hispanic COPD patients; nonprofessional caregivers; experts in health and social disparities; clinicians (geriatrician, pulmonary expert, and a respiratory therapist); and patient advocates. The role of the CAB was to advise the study team on all aspects of study design, implementation, evaluation, and dissemination over time. More specifically, the CAB was responsible for identifying factors expected to impact acceptance and feasibility among this population. Although feedback from all CAB members was incorporated, the study team gave particular weight to patient stakeholder feedback. Discussions lasted around 2 hours each and were audio recorded and then professionally transcribed.

### Data Analysis

Content analysis was used to analyze transcript data. After each session, the recordings were professionally transcribed, and a facilitator developed a codebook that allowed for categorization of the data. Moreover, 2 qualitative researchers initially worked independently on the coding of each transcript. After completion of the coding, the researchers met to discuss and revise the codes. A process of discussion and reflection was used to settle any disagreements on the codes. Thereby, researchers were able to identify themes and relationships found in the data [25,26]. The main themes that emerged identified specific recommendations and perceptions of barriers and facilitators for intervention implementation.

#### Ethics, Consent, and Permissions

All study activities were approved by the institutional review board (#16-663; Feinstein Institute for Medical Research’s Institutional Review Board). All participants consented to study participation and audio recording.

#### Interventions

The standard pulmonary rehabilitation (SPR) arm receives a referral to SPR, which occurs in a rehabilitation center and meets twice a week for 8 weeks. The SPR arm also receives an automatic linkage to a social worker to navigate health care resource access and manage social work–related concerns. Patients in the telerehabilitation arm receive a referral to comprehensive pulmonary disease management program (CPDMP) for 8 weeks. CPDMP is identical to the SPR comparator except for the delivery of PR being via telehealth in either the patients’ home or community center (depending on their preference). Blood pressure, oxygen saturation rate, and pulse or heart rate are measured before, during, and after sessions and transmitted in real time to the respiratory therapist conducting the sessions.

During the first CAB meeting, a general discussion of community needs was followed by a dialog regarding specific factors about both intervention equipment and study design. CAB members were presented with the intervention (in English and Spanish), and a demonstration of core components and vital adjustable characteristics of the intervention was provided. A qualitative consultant led the community discussions, with content guided by predetermined topics outlined in an interview guide, including instructions to prioritize patient stakeholders’ contributions above medical and professional stakeholders’ contributions [27]. The first meeting (held before the start of the RCT) focused on adapting the intervention and included a discussion of (1) ease of intervention use, (2) intervention usefulness, (3) barriers to intervention implementation, and (4) adjustment recommendations. A second meeting was held with the CAB (also, pre-RCT) to present and confirm adaptations made to the intervention based on the CAB’s recommendations. All meetings were conducted in both English and Spanish with a professional translator in attendance.

## Results

### General Themes and Subthemes

The results described below show the general themes and subthemes that arose from the 2 focus groups of the CAB stakeholder meetings. The discussion centered around the adaptation of the PR TM program, from both consumer and provider perspectives. Consumer perspectives mainly focused on cultural tailoring, whereas provider perspectives focused on equipment functionality (eg, Can a frail, older patient sit on the bike?).

The first meeting began with a general discussion of the needs of COPD underserved patients; multiple challenges were identified, including factors that affect access to PR. These factors included insurance payment for PR, repeated hospitalization, medication management, and comorbidities.

After the general needs assessment discussion, the principal investigators presented a demonstration of the initial version of the rehabilitation equipment (a bicycle). The equipment demonstration illustrated that the equipment initially overwhelms some patient stakeholders; although the hands-on aspect of the demonstration helped them feel more at ease, the CAB felt that important adaptations were needed to ensure safety and improve the usability of the intervention.

### Theme 1: Equipment Changes

#### Subtheme 1: Safety and Comfort

One theme that emerged quickly was bike ergonomics: the need for a bike that was safe and comfortable for patients with relatively limited mobility. Specifically, 1 participant noted that the bike would need to be stable with a seat that patients can get in and out of easily. One patient noted:

You don’t have to struggle... with all the equipment, everything is set up just right for the patient to access everything.

Specifically, the upright nature of the bike was perceived as dangerous, as patients could more easily fall because of the unstable nature of the design; a recumbent bicycle was strongly encouraged by the CAB. A patient noted:

The bike is not difficult, but the bike was breaking down. The arm wasn’t good...

Another provider participant noted that the tablet attached to the bike was too high and needed to be at eye level. The arm, which holds the tablet, should have the ability to *swivel* to facilitate getting in and out of the bike and to accommodate for varying patient heights. A stable arm or surface was deemed necessary to help with transfer; choosing the voice that speaks to the patient, in the accent that is most familiar; considering the gender of the patient and their wardrobe (wearing dresses); and a wider seat with more cushioning than a typical bike to accommodate an older, larger body.

#### Subtheme 2: Ability to See Vital Signs

One patient participant discussed that he would want the ability to see his vital signs while exercising on the bike. Having access to feedback—the ability to look at vital signs—is a good teaching opportunity for participants. If patients make adjustments during exercise and see an improvement in their vitals, this feedback reinforces what they are being taught by the respiratory therapist.

### Theme 2: Recruitment Changes

#### Subtheme 1: Recruitment Brochure

CAB patient participants recommended multiple updates to a recruitment brochure that was developed by staff. These changes included increasing the font size, testing the grade level it is written in, and checking that information is accurately translated for multiple dialects. For example, several patient members of the CAB suggested not just using the term *COPD* but also using the term *Enfermedad pulmonar obstructiva crónica* (*EPOC*).

#### Subtheme 2: Culturally Tailoring the Recruitment Process

A second theme that emerged from the discussion was the need to culturally tailor the enrollment process for a population that does not often receive PR or frequently participates in research. At the third CAB meeting, the staff presented a recruitment video and a recruitment brochure in response to previous CAB recommendations to develop a recruitment video. The video used an untrained actor posing as a patient. One patient suggested including multiple patients exercising together in the video. This would show communication and support among patients. Another patient suggested having real patients in the recruitment video (as opposed to untrained actors) to make the experience look more realistic:

...the video was very good, but I think it will be much better when they get more people involved...they’re communicating together at the same time, it’s like...supporting each other, you know...

It didn’t impress me much. I liked the part that you played, I thought you did a very good job there. But he doesn’t really look the part...

I think you want to think about incorporating the caregiver, because...it’s not just the patient... it’s the spouse (Mrs Lopez)( *names changed to protect patient privacy) who’s been watching... been part of the story of Mr. Lopez, just as much as he’s been a part. Different role, but together. [Mrs. Lopez] was very clear and very eloquent talking about this. And... [Mrs Lopez] said that now you can’t get him off the bike... It’s an inspiration to see Mr. Lopez, but also to hear Mrs. Lopez...So I would just suggest you think about the caregiver.

Study enrollment was discussed at the second CAB meeting; both patients and providers suggested using a recruitment brochure and a video for enrollment that shows a patient with COPD that looks like them (Hispanic and African American patients) successfully participating in PR.

### Theme 3: Study Logistics

#### Subtheme 1: Lateness Protocol

The respiratory therapist asked the CAB what to do when a patient arrives late to the multiuser session. Both patients and providers suggested having a protocol in place for when this happens, including putting a time limit on how late they can arrive, stating that they will not receive extra time if they are late, or providing phone call reminders before the start of class:

When you’re on a multi-person call... if people start showing up late, everything gets disrupted, so now you’ve got a dosage issue because your one-hour session just became 45 minutes because Person 1 was there on time, Person 2 was ten minutes late...and you’ve got to redo your greetings, and you—it disrupts everything.

#### Subtheme 2: Gradual Exercise

The CAB discussed the patient educational process in using the bike for exercise. Specifically, providers recommended that the exercise be presented as graded tasks (ie, starting simple with 1 or 2 instructions and adding on over time). Given that the COPD bike is different from an ordinary bike, requiring an extended, longer, and more gradual learning period, it is essential for patients to be comfortable with what they are doing and understand what is going on:

Exactly, you cannot just throw it at one time, you gotta educate them, like from A to B,... you gotta teach them step by step.

### Theme 4: Self-Efficacy

#### Subtheme 1: Communication

The committee discussed the importance of being able to communicate effectively during exercise. This communication between both the respiratory therapist and patient is to foster learning and understanding of the program and how patients are reacting to the movements. Our patient partner shared:

Yes, absolutely. I am able to hear him loud and clearly, we’re able to interact and switch between bike and exercises and warm-up, and I ask him to (put on his?) blood pressure cuff and his pulse oximeter, everything is—we are able to do pretty much everything.

#### Subtheme 2: Motivation

One participant discussed how motivating it is for them to see others succeeding on the bike, particularly patients who are similar in terms of age, condition, ability, etc. He noted that seeing others succeed gives him the confidence that he too can successfully participate in the program. Another patient commented on how they were personally motivated to do more things because of the program:

The way [Mr. Lopez] here was doing the bike, when we see the video, we get motivated seeing him doing it...the way he’s breathing is excellent according to what I saw there.

It got me out of my room with my anxiety and my depression—it motivated me to do other things that I didn’t do before.

#### Subtheme 3: Health Control

There was additional CAB discussion about regaining control of one’s health resulting from perceived improvements in health status as a result of exercise. The discussion reflected that patients have a *better understanding* of what to do to improve their health. Patients who completed the program commented that they were able to see improvements from the exercise and use what they have learned outside of the program:

I liked them a lot, I wanted to see if this worked for me. It wasn’t easy at first because I wasn’t used to it, but now I can even do them on my own.

It was hard in the beginning. Because when you don’t exercise for a long time and then you start—it is stressful but then gets easier. I was always allowed to rest during the sessions. I felt an 80% improvement. My chest isn’t that tight and I can breathe through my nose when I do the breathing exercises that I learned in the program.

#### Subtheme 4: Importance of Coaching and Presence in the Home

The final subtheme that emerged from the CAB meetings came from our patient advocate, who indicated that, given the importance of culturally congruent social interaction within the Hispanic community, having a combination of in-person interaction in addition to Web-based interaction (via technology)—rather than exclusively online—is more meaningful and preferable to patients.

### Theme 5: Access

On multiple occasions, the CAB discussed the benefit of having access to PR right in one’s home. Given that for this patient population, it is not always feasible to travel to therapy, having this access allows patients to continually participate in exercise—an important part of maintaining quality of life. One patient also noted that they felt more comfortable being at home with this program than going out to a gym:

The other thing I think it’s great, because in the home...winter time, it’s snowing, raining, sleet, and all that stuff... sometimes it’s not feasible to get there. So, if you got [the respiratory therapist] on the screen, you can still do what you got to do.

## Discussion

### Principal Findings

Although previous meta-analyses have documented the clinical efficacy of remote monitoring of patients with chronic illness [28,29] and discussed the importance of adapting interventions to facilitate cultural relevance [17,18], this is the first study to describe the formative process of a CBPR study aimed at optimizing home TM utilization among African American and Latino COPD patients from underserved communities. As noted above in detail, 5 major themes emerged from qualitative analyses of the CAB meetings. Interestingly, although some of the tailoring was *cultural* in nature (eg, developing recruitment brochures and videos of persons who *look like us*), much of the tailoring had to do with intervention comfort and logistics. For example, progressive teaching to self-efficacy was an important theme that transcends culture and language.

### Intervention Changes

Several significant changes to the equipment were made in response to CAB recommendations. Specifically, the CAB recommended lowering the height setting on the bike to accommodate patients who may be unsteady *climbing up* onto the seat. To ensure patient safety in getting on and off the equipment and to ensure the patient’s competency in using the equipment, the CAB recommended that the patient be accompanied by a staff person for at least the first session.

Another intervention adaptation involved welding the arm that holds the pulse oximeter to the back of the bike to remove restriction of movement because of the wire from the pulse oximeter. Similarly, a second arm was welded onto the bike that allowed the patient to move the tablet to the best possible position for viewing.

Other intervention adaptations to facilitate safety included the replacement of the pulse oximeter by a Nonin watch, whereby sensors are kept on throughout the whole session and sensor values are directly sent to the respiratory therapist for immediate monitoring. Tablets were also adapted for icon use to facilitate easier access to different aspects of the app (*point and click*), regardless of patient primary language.

Protocols for handling patients who signed on to the session late (thereby interrupting others) were also set by the CAB. Similarly, the CAB also suggested the importance of graded tasks, or mastery experiences, as an important component of self-efficacy: in this case, having the respiratory therapist gradually introduce exercises to patients over time to facilitate task confidence.

### Recruitment Changes

CAB changes to the recruitment process included the use of a recruitment brochure and a video that presents real patients *that look like us* (vicarious modeling) rather than actors. Brochures were developed to inform prospective patients about the study and leave detailed information with the patient to share with caregivers and family. The CAB also strongly suggested utilizing a common language rather than medical jargon. For example, using the term *EPOC* was recommended to accompany the term *COPD*, which is often unfamiliar to Hispanic patients without medical backgrounds and can be especially daunting to those patients with lower health literacy and for whom English is a second language. The CAB also suggested that the recruitment team work with inpatient respiratory therapists to help create *buy in* from hospital staff to recruit for the study.

In addition, as a result of CAB recommendations, recruitment staff were trained in motivational interviewing techniques. Finally, in response to recruitment challenges, the CAB suggested that we expand recruitment efforts from inpatient to outpatient clinic recruitment as long as the patients met the criterion of no more than 3 weeks from hospital discharge.

### Study Limitations

This qualitative study limited data collection to a CAB with membership from the New York metropolitan area. Thus, findings from the study may not be generalizable to other settings and should be interpreted with caution. Other study limitations include the small number and mixed nature of the groups, potentially limiting the likelihood that full theme saturation was reached.

The strength of this qualitative study is the utilization of a CBPR approach. The role of the CAB was to provide the study team with stakeholder perspectives and guidance in the development, implementation, and evaluation of home TM COPD rehabilitation intervention tailored to underserved African American and Hispanic patient populations. The CAB led discussions on adaptation, usability, and program satisfaction and ensured that the conduct of the research remained patient-oriented. Each member of the CAB provided a unique perspective. All members had different expertise, skills, and experience, which contributed to the success of the adaptation of the intervention. As evidenced by our themes, the CAB was critical in identifying and resolving issues that surfaced during the project. A CBPR process was necessary to ensure that relevant cultural and patient-centered factors were addressed in optimizing our intervention as feasible and acceptable to African American and Hispanic underserved patients.

CAB meetings were held in English and in Spanish simultaneously through the use of a live interpreter. Although the translation resulted in a slower session, it facilitated the inclusion of stakeholder perspectives from both English- and Spanish-speaking participants.

### Conclusions

There has been a dearth of literature regarding telehealth adaptation in COPD patients from underserved communities. This qualitative study allowed us to gauge community stakeholder perspectives about intervention adaptations for PR at home. Identifying adaptations that are important to key stakeholders through the CBPR-based method is a necessary process to ensure that a complex intervention is generalizable for patients from underserved communities.

## Appendix

Multimedia Appendix 1 Moderator guide.

## Abbreviations

CAB: community advisory board
CBPR: community-based participatory research
COPD: chronic obstructive pulmonary disease
CPDMP: comprehensive pulmonary disease management program
EPOC: Enfermedad pulmonar obstructiva crónica
PCORI: Patient-Centered Outcomes Research Institute
PR: pulmonary rehabilitation
RCT: randomized controlled trial
SES: socioeconomic status
SPR: standard pulmonary rehabilitation
TM: telemonitoring
