# Supplementary material for: A Telehealth-Delivered Pulmonary Rehabilitation Intervention in Underserved Hispanic and African American Patients With Chronic Obstructive Pulmonary Disease: A Community-Based Participatory Research Approach
Source: JMIR Form Res. 2020 Jan 31;4(1):e13197. doi: 10.2196/13197 (PMC7055744; doi:10.2196/13197)
Supplement: Multimedia Appendix 1 [file formative_v4i1e13197_app1.pdf]

Community Advisory Board (CAB) Focus Group Discussion Guide

INTRODUCTION

Good afternoon. Thank you for participating in this focus group. My name is [redacted] I will be facilitating the discussion today. We are interested in learning about your experiences and opinions on the telehealth program for COPD patient pulmonary rehabilitation. There are no right or wrong answers to the questions. We are interested in hearing your opinions, perspectives and personal experiences that will help us to adapt the intervention and make it as useful as possible for Latino COPD patients. We ask that everyone share their opinion and talk one at a time. We really appreciate your help.

Remember, your answers are confidential. No one outside this room will know what you, in particular, said because your name will not be associated with your responses. We are asking that you sign the consent form before we begin that indicate your agreement 1) to be recorded; and 2) to allow your responses to be included in our study.

The conversation will be audio recorded so that my research team and I can listen to it and transcribe it later. The recording will be kept safely in a locked facility until it is transcribed word for word, then it will be destroyed. The transcribed notes of the focus group will contain no information that would allow individual people to be linked to specific statements. Remember, your participation is voluntary and you are free to stop participating at any time.

ICE BREAKER – self introductions

There are **<u>three</u>** ways that you can contribute to today's discussion:

1. You can share your personal experience (again, we remind you that what is said here today will remain confidential and anything you share won't be linked back to you).
2. You can also share your thoughts and ideas as they pertain to Latino patients with COPD. For instance, you can talk about someone that you know or that you have heard about (please don't use their names, refer to them as "a friend").
3. You can share your thoughts about any adjustments that need to be made to the pulmonary rehabilitation telehealth program so that it is most relevant and appropriate for use by the target patient population.

These comments will help us to understand how to better help patients with COPD and reduce the risk of future hospitalizations.

Remember today that **<u>you</u>** are the expert and we are the students!

<u>Do you have any questions about the study or the interview before we get started?</u>

<u>May we turn on the audio recorder now?</u>

Background experience, description of the patient population and research participation

#	Questions	Probes
1	First, let's take a moment to talk about our own experiences with COPD, as patients, providers or health advocates from the community.	• Number of years<br>Patients<br>• Hospitalization<br>• Recent?<br>• How many times?<br>• Number of ED visits?<br>• Diagnosis<br>• Treatment<br>• Current health status in relation to COPD

		Providers<br>• Training/work experience<br>• Community advocates
2	How would you describe most patients with COPD in the Latino community?	• Gender<br>• Race/Ethnicity<br>• Age<br>• Marital status<br>• Employment status<br>• Education<br>• Household composition<br>• Social support<br>• Insurance<br><br>Clinical issues: Co-morbidities<br>• Hypertension<br>• Asthma<br>• Sleep apnea<br>• Depression or other mental health issue<br>• Chronic inflammation<br>• Fractures<br>• Hep B or C<br>• HIV infection<br><br>Life-style<br>• Alcohol and drug use<br>• Smoking<br><br>Care & Treatment<br>• Where do they go for care and treatment?<br>• General provider vs. specialist<br>• How often do you go for care<br>• Multiple sites<br>• Recent hospitalizations<br>• ER visits<br><br>Barriers to treatment<br>• Transportation (distance or difficult to access)<br>• Money (cost of getting to visits; have to work)<br>• Caregiver responsibilities (for children, parents or sick partner)<br>• Concerns about privacy or disclosure
3	What do you think are the most important treatment needs for patients with COPD in this community?	• Access issues<br>• Provider issues<br>• Reduction of hospitalizations<br>• Self-care<br>• Quality of life
4	What do you think are barriers to participation in research in this patient population?	• Mistrust<br>• Breach of confidentiality<br>• Documentation status
5	What are your feelings about pulmonary rehabilitation?	• Purpose?<br>• Do you think it is beneficial?
6	What has been your experience with pulmonary rehabilitation?	• How many times?<br>• What was your experience?

7	What suggestions would you make about reducing any barriers to pulmonary rehabilitation?	• Transportation<br>• Cost<br>• Time<br>• Discomfort

Demonstration of the Telehealth Bike (25 min)

Now we are going to take some time to demonstrate how to use the telehealth bike as if you were a potential patient. We will have time at the end to answer any questions you may have. We will also ask you questions about how we can improve our training methods.

NOTE: Provide as much time as is needed to address any questions. When appropriate, move into the next section.

Assessment of Telehealth Bike and Training

Thank you for your questions.

Now we would like to ask some questions about the telehealth program so that we improve our methods of training or address potential barriers to its use. These questions will help us identify how we might need to modify the telehealth program so that it is appropriate for Latino patients with COPD in this community.

8	First, let us ask: What are your initial impressions of the program?	• Positive or negative impressions<br>Follow up question:<br>• Is there anything about the telehealth program that makes you uncomfortable?
9	After this demonstration, do you think that you would be able to use the telehealth program?	Probe:<br>• Positive or negative impressions<br>• Barriers<br>• What could make it challenging for patients to use the telehealth bike?<br>Do you think that other Latino patients with COPD in this community will be able to use it?<br>• Positive or negative impressions<br>• Barriers<br>• What could make it challenging for patients to use the telehealth bike?
10	How easy do you think it will be to learn to use this equipment?	• Positive or negative impressions
11	Were the instructions clear?	• Points of confusion or clarity<br>• Was there too much jargon?<br>• Were the Spanish translations accurate?
12	Was the demonstration helpful?	• Points of confusion or clarity
13	How do you think the telehealth program can affect health and wellbeing for Latino patients with COPD?	• Positive or negative impressions<br>◦ Empowerment<br>◦ Improve self-efficacy<br>◦ Create feelings of alienation<br>◦ Create feelings of suspicion<br>Follow up questions

		• How can the telehealth pulmonary rehabilitation program affect Latino patients' ability to manage their health?
14	How easy do you think it will be for Latino patients to use the telehealth program?	• What environmental or social variables could make it difficult for patients to use the bike?
15	Does the equipment seem easy to use?	• Is the screen large enough?<br>• Is the font large enough?<br>• How easy did it seem to use the screen?
16	How easy did it seem to talk to the medical professional during a video conference? Feelings about having someone see you in your home?	• Positive or negative impressions
17	Do you think that Latino patients will feel that the experience of talking with the medical professional during the telehealth session will be as satisfying as talking to them in person?	• Why?
18	Do you feel that Latino patients will be concerned about their privacy when talking with the medical professional during the telehealth session?	• Why?<br>• If yes, suggestions?
**Technological concerns**<br>Now we would like to ask you specific questions about potential problems that Latino patients might experience with the technology used in the Pulmonary Rehabilitation Telehealth program		
19	After today's presentation, do you think that the technology in pulmonary rehabilitation telehealth program is "user-friendly" for patients in our community?	• Do you think that individuals intimidated by smart technology will be reluctant to use telehealth?<br>• Do you think that many will think this is too "high-tech" for them?<br>• Do you think that it might seem too complicated or confusing?<br>• How many patients do you think are technology savvy?
20	Do you think patients in this study will have any problems with the following:<br>• Size of the screen<br>• Touch-screen technology<br>• Size of the font<br>• Location of the buttons<br>• Using any of the medical equipment<br>• Reading the results from equipment	• Probe for specific problems and ideas for how to address or fix these potential issues<br>• Probe for attention to special needs (e.g., visual or hearing disability, extreme arthritis, limited mobility, etc.)
**Language Issues**<br>Now we would like to ask you some questions about the language used in the training and on the Pulmonary Rehabilitation Telehealth program.		
21	Was the language used clear and concise?	• Was there too much medical jargon?

22	How could we improve how we describe the product or talk about Telehealth in general?	• What would be a better way?
23	We have also translated all of the materials into Spanish. For those of you who are Spanish-speakers, could you help us determine if the Spanish translations are accurate?	• Are the translated screens in Spanish in the right dialect for our Spanish-speaking population?<br>• Are the Spanish translated screens similar or different from the English version?<br>• How important will it be for the patient population to also have the medical professional speak a Spanish dialect that matches the patient population? How difficult is it to understand different dialects?<br>• Which national dialects are most common among our patient populations?<br>◦ Puerto Rico<br>◦ Dominican Republic<br>◦ Mexico<br>◦ South America (Venezuela, Columbia, Equator, Argentina, Etc.)<br>◦ Central America (Honduras, Costa Rica, El Salvador, etc.)

Potential environmental issues

Now that you have had an opportunity to see how the telehealth bike is used, we want to ask you about some of the environmental issues in the home that may or may not make it difficult for patients to use the Pulmonary Rehabilitation Telehealth program. These questions pertain to time limitations and social support.

24	Another possible barrier to using the Telehealth program is not having enough time to conduct the sessions. We anticipate that some patients will have busy lives and may not have enough time. Do you think that patients may have problems making a time commitment for the telehealth pulmonary rehabilitation sessions?	• How can we help busy patients fit the telehealth sessions into their schedule?<br>• Caregiver schedules<br>• Patient schedules<br>• Are the sessions scheduled at a convenient time
25	We know that having social support can be essential to treatment adherence. Do you think that having someone other than the patient is necessary to help them use the Telehealth program?	• What kind of social support is ideal?<br>• What should be done to help patients that do not have social support?<br>• Finally, many patients may have health aides to help them in the home. Many of these health aides work with patients for short periods of time.<br>• How can we help patients educate their new health aides on the Telehealth Pulmonary Rehabilitation program so that they can continue to get help when they need it?
26	Did you have any comments or suggestions for how to help Latino patients with COPD to use the	• Training<br>• Language used<br>• Help with use of the bike

	Telehealth Pulmonary Rehabilitation program?	
27	How would the telehealth program help to reduce barriers to pulmonary rehabilitation?	• Transportation<br>• Cost<br>• Time<br>• Discomfort
	**Final comments or suggestions**<br>We've reached the end of the discussion. Do you have anything else that you would like to add about the topics that we have discussed here today?<br><br>**THANK YOU FOR YOUR TIME AND FOR YOUR IMPORTANT INPUT!**	• Of all the things we have discussed today, what would you say are the most important issues you would like to express about the telehealth pulmonary rehabilitation program?
